# O1-8 Evaluation of the implementation of the ‘Living well with COPD' self-management program in Switzerland

**DOI:** 10.1093/eurpub/ckac094.008

**Published:** 2022-08-29

**Authors:** Anja Frei, Alexandra Strassmann, Mathias Guler, Tania Carron, Claudia Steurer-Stey, Kaba Dalla Lana, Philippe Giroud, Isabelle Peytremann-Bridevaux, Milo Puhan

**Affiliations:** 1 Epidemiology, Biostatistics and Prevention Institute, University of Zurich, Zurich, Switzerland; 2 Swiss Lung Association, Berne, Switzerland; 3 Centre for Primary Care and Public Health (Unisanté), University of Lausanne, Lausanne, Switzerland

**Keywords:** Self-management program, exercise capacity, implementation evaluation, chronic disease, chronic obstructive pulmonary disease

## Abstract

**Background:**

Self-management can improve health status and reduce hospitals admissions in patients with chronic obstructive pulmonary disease (COPD). The aim of this mixed-methods study was to evaluate the implementation of the ‘Living well with COPD’ self-management program, a nation-wide effort by the Swiss Lung Associations and the Swiss society of pulmonary medicine.

**Methods:**

For the implementation evaluation we used qualitative (interviews, focus groups) and quantitative (questionnaires, documentation analysis) methods to assess indicators of the outcomes reach, dose, fidelity, acceptability and appropriateness of the program. To evaluate the effectiveness, we assessed exercise capacity (1-minute sit-to-stand test), disease specific quality of life (Chronic Respiratory Questionnaire CRQ), symptoms, health care utilisation and health behaviour including physical activity at baseline and at the end of the program (after 12 months).

**Results:**

Seven Cantonal Lung Associations implemented the program into their services according to plan, conducted it 13 times during 1 year and included in total 122 COPD patients (mean age 69 years, 48% female). The patients' attendance rate and the coaches' fidelity to the protocol were high (81% and 94%, respectively). Overall, acceptance and satisfaction of all involved persons (patients, coaches, responsible persons from the Lung Associations) was very high; they particularly acknowledged the meaningfulness of the program. Challenges were sustainable funding, integration of the coaches' additional workload and uncertainties regarding roles and responsibilities. After 14 month, the patients did not just avoid a decline in exercise capacity and health status but significantly improved in the 1-minute sit-to-stand test (23.9 vs. 27.1 repetitions) and in 3 of 4 CRQ subscales (with 0.2-0.5 units).

**Conclusions:**

The ‘Living well with COPD' program was successfully implemented despite a tight schedule and showed a remarkably positive impact of the COPD patients' health status. The insights of this study will support the broader multiplication of the program throughout Switzerland and also serves the international community since it is one of the first nation-wide implementations beyond study settings.

